# Quantifying the Functional Gap in Alkaptonuria Through Machine Learning and Clinical Data Integration

**DOI:** 10.3390/bioengineering13060604

**Published:** 2026-05-22

**Authors:** Anna Visibelli, Rebecca Finetti, Bianca Roncaglia, Alfonso Trezza, Barbara Marzocchi, Ottavia Spiga, Annalisa Santucci

**Affiliations:** 1 Department of Biotechnology, Chemistry and Pharmacy, University of Siena, 53100 Siena, Italy; anna.visibelli2@unisi.it (A.V.); rebecca.finetti@student.unisi.it (R.F.); bianca.roncaglia@unisi.it (B.R.); alfonso.trezza2@unisi.it (A.T.); barbara.marzocchi@unisi.it (B.M.); ottavia.spiga@unisi.it (O.S.); 2 UOC Laboratorio Patologia Clinica, Ospedale S. Maria alle Scotte, AOU Senese, 53100 Siena, Italy; 3 Centro della Scienza e della Tecnica, Polo Universitario Grossetano, Via Ginori 41, 58100 Grosseto, Italy; 4 Industry 4.0 Competence Center ARTES 4.0, Viale Rinaldo Piaggio, 56025 Pontedera, Italy; 5 MetabERN, Department of Biotechnology, Chemistry and Pharmacy, University of Siena, Via Aldo Moro, 53100 Siena, Italy

**Keywords:** alkaptonuria, functional gap, rare diseases, machine learning, data integration, precision medicine

## Abstract

Alkaptonuria (AKU) is a rare inherited metabolic disorder characterized by progressive musculoskeletal damage, chronic pain, and functional heterogeneity. To better quantify this variability, we introduced the concept of the functional age gap, defined as the difference between chronological age and a data-derived estimate of functional age. The study included 134 patients with AKU from the ApreciseKUre database. Functional age was calculated by mapping Health Assessment Questionnaire Disability Index (HAQ-DI) and Knee Injury and Osteoarthritis Outcome Score (KOOS) values to age-referenced normative data. Most patients (94.8%) showed a positive functional age gap, with a mean difference of 15 years, which indicates a functionally older profile than expected for their chronological age. A bagging ensemble of decision trees was then used to explore relationships between clinical variables and functional age gap severity. The model achieved moderate but stable classification performance across repeated stratified cross-validation (64%), consistent with an exploratory analysis in a small rare-disease cohort. SHapley Additive exPlanations analysis identified age, AKUSSI spinal pain, AKUSSI joint pain, Schober test, and hip and knee activity as the most influential predictors. These findings support the functional age gap as an interpretable, hypothesis-generating descriptive metric for functional assessment in AKU, while its predictive utility for individual patient stratification will require validation in larger and longitudinal cohorts.

## 1. Introduction

Alkaptonuria (AKU) is a rare inherited metabolic disorder caused by deficiency of the enzyme homogentisate 1,2-dioxygenase (HGD), leading to systemic accumulation of homogentisic acid (HGA) [1,2,3]. This metabolic alteration results in a progressive, multisystem disease characterised by ochronosis, the deposition of HGA-derived pigment in connective tissues, and a severe form of early-onset osteoarthropathy [4,5,6,7,8,9]. In addition to dark urine, ochronosis, and joint degeneration, AKU is associated with a broad spectrum of clinical manifestations, including renal and prostate stones, cardiovascular complications, bone fragility, and tendon or ligament damage, reflecting its systemic nature [10,11]. Although AKU is present from birth, its clinical manifestations typically emerge later in life. The disease course is highly heterogeneous, with substantial inter-individual variability in symptom onset, severity, and progression [12]. Notably, this variability cannot be fully explained by genetic determinants alone, suggesting the involvement of additional clinical, metabolic, and biomechanical factors [13,14]. This intrinsic heterogeneity, combined with the rarity of the disease and the non-specific nature of early symptoms, complicates diagnosis, monitoring, and prognostic assessment. Functional impairment is one of the most clinically relevant aspects of AKU. Progressive musculoskeletal involvement, primarily impacting the spine and major supportive joints, results in chronic pain, restricted mobility, and progressive disability, which compromises patients’ independence and quality of life (QoL) [15]. QoL is a multidimensional construct including physical, psychological, and social functioning as perceived by patients. In chronic and rare diseases, QoL is a particularly relevant outcome because it captures dimensions of disease burden that are not fully reflected by clinical or biochemical markers alone. Consistently, a recent systematic review on health-related quality-of-life scales in rare diseases highlighted the heterogeneity of available instruments and the difficulty of comparing patient-reported outcomes across cohorts [16]. In this context, QoL measures have been proposed as valuable tools to assess health needs and evaluate disease burden in AKU [17,18]. Its assessment is, however, inherently challenging, especially in rare conditions, where marked heterogeneity in disease progression, symptom severity, and individual adaptation may lead to substantial variability in patient-reported outcomes [19]. To quantify disease burden, several clinical and patient-reported outcome measures are employed, including the Health Assessment Questionnaire (HAQ) [20], the Knee Injury and Osteoarthritis Outcome Score (KOOS) [21], and AKU-specific composite indices such as the Alkaptonuria Severity Score Index (AKUSSI) [22,23,24]. Among these, HAQ is widely used to assess disability in daily activities, whereas KOOS is a knee-specific patient-reported outcome measure that covers pain, symptoms, activities of daily living, sport and recreation, and knee-related quality of life. However, these instruments capture different dimensions of functional status, which are not always consistent with one another or with the severity of the underlying disease. In particular, discrepancies are often observed between objective indicators of disease progression and patient-reported outcomes, suggesting that standard scoring systems do not fully capture all relevant aspects of functional impairment [25]. In recent years, the increasing availability of structured clinical datasets has enabled the use of artificial intelligence (AI) and data science in the study of rare diseases [26]. Despite the challenges posed by small sample sizes and fragmented data, machine learning (ML) approaches have demonstrated their effectiveness in identifying meaningful patterns in diverse data sets [27,28]. This has led to advancements in patient stratification and the identification of clinically relevant features. In the context of AKU, these efforts have been supported by the development of the integrated data platforms ApreciseKUre, a comprehensive digital ecosystem designed to collect and harmonize genetic, biochemical, clinical, histopathological, and quality-of-life data from patients with AKU [29,30,31]. This resource has enabled the application of statistical and ML tools to uncover previously unrecognized correlations, support patient stratification, and explore genotype-phenotype relationships, thereby advancing the implementation of precision medicine approaches in this rare disorder. Nevertheless, despite these advances, a methodological gap remains in the integration and interpretation of multiple functional assessments.

Although the AKUSSI, HAQ-DI and KOOS questionnaires are well-established tools for quantifying disease burden in AKU, they each capture only a partial dimension of patient status and report it on a scale that is not directly interpretable in terms of biological or functional ageing. AKUSSI reflects AKU-specific clinical severity, HAQ-DI measures general disability in daily activities, and KOOS provides knee-specific patient-reported outcomes [32]. Reporting on these instruments independently therefore provides an incomplete picture of patient burden and does not permit direct comparison of the functional status observed in a given patient with that expected for their chronological age. To address this limitation, we introduce the concept of the functional age gap, defined as the discrepancy between chronological age and a data-derived estimate of functional age. In this study, we use a data-driven approach to quantify and interpret the functional gap in AKU. Through the integration of clinical and functional data, we aim to systematically quantify discrepancies across functional measures, examine their association with clinical and demographic variables, and identify the key determinants underlying inter-individual variability.

## 2. Materials and Methods

### 2.1. Dataset Description

This study was designed to quantify functional impairment in patients with AKU through the definition of a functional age and the estimation of a functional age gap. The analysis was performed on the ApreciseKUre database, an integrated patient-level dataset including demographic, anthropometric, biochemical, clinical, and patient-reported outcome variables. The dataset included sex, year of birth, body mass index (BMI), serum amyloid A (SAA), chitotriosidase, advanced oxidation protein products (AOPP), PSH, CySSP, CyGlySSP, HcySSP, GluCySSP, GSSP, RSSP, and protein thiolation index (PTI), as well as clinical severity indicators such as AKUSSI joint pain, AKUSSI spinal pain, Schober test, mean hip activity, and mean knee activity. Functional status was assessed using the Health Assessment Questionnaire Disability Index (HAQ-DI) and the five Knee injury and KOOS subscales, namely pain, symptoms, activities of daily living, sport/recreation, and quality of life (see Table S1 for detailed descriptions). Patients were included if they had a confirmed diagnosis of alkaptonuria, were registered in the ApreciseKUre database, and had the chronological age, HAQ-DI, and KOOS data required to compute the functional age gap. Patients were excluded only when age or functional data were missing/inconsistent, or when clinical records were too incomplete to be harmonized within the analytical dataset. No exclusions were applied based on sex, disease severity, treatment status, or biochemical profile, to preserve the representativeness of this rare-disease cohort.

### 2.2. Functional Age Gap

Functional age was derived from HAQ-DI and KOOSs, mapping each patient’s value to the closest age category provided in the corresponding normative reference tables [33,34]. For each questionnaire, the reference tables report a single representative value (population mean) for each decadal age category (30–39, 40–49, 50–59, 60–69, 70–79, and 80–89 years). For each patient, the assigned age category was the one whose reference value had the minimum absolute distance from the observed score, evaluated over all reference values available for that questionnaire. When a patient value fell outside the range spanned by the reference table, the algorithm assigned the patient to the nearest extreme category, which corresponds to an implicit clipping at the boundaries of the normative distribution. Each assigned category was then converted into its midpoint value (34.5, 44.5, 54.5, 64.5, 74.5, and 84.5 years, respectively). The HAQ-derived functional age was obtained directly from the HAQ-DI midpoint. The KOOS-derived functional age was computed as the arithmetic mean of the midpoints obtained from the five KOOS subscales, so that each subscale contributed equally to the integrated knee-related estimate. Finally, the overall functional age was defined as the arithmetic mean of the HAQ-derived and KOOS-derived functional ages, giving equal weight to the two instruments, and the functional age gap was computed as the difference between functional age and chronological age. Positive values indicated a functionally older profile than expected for chronological age, whereas negative values indicated a functionally younger profile.

### 2.3. Dataset Preprocessing and Model Implementation

All preprocessing, statistical analyses, and predictive modeling steps were implemented in Python (version 3.11). The variable with the highest proportion of missing values was mean knee activity, at 7.46% (10 out of 134 patients). This was followed by KOOS sport (5.97%) and GluCySSP (5.22%). All other variables had a missing value proportion of ≤4%. Overall, 102 patients (76.1%) had complete information across all variables involved in the analysis. As no single variable exceeded the 10% threshold, missing values were handled by mean imputation, and continuous variables were normalized with min-max scaling.

For the classification task, the 134 patients were distributed across the three functional age gap severity classes as follows: class 0 (gap < 11.5 years), *n* = 44 (32.8%); class 1 (11.5 ≤ gap < 18.5 years), *n* = 40 (29.9%); and class 2 (gap ≥ 18.5 years), *n* = 50 (37.3%). The three classes were therefore approximately balanced, with each class accounting for between 29.9% and 37.3% of the cohort.

A multiclass classification model was then developed using demographic, biochemical, and clinical variables as predictors. Several candidate classification models, including simpler baseline approaches, were evaluated during the optimization phase. Hyperparameter optimization was performed by grid search using classification accuracy as the selection metric. Among the tested models, the bagging ensemble of decision tree classifiers showed the best balance between classification performance and cross-validation stability and was therefore selected as the final model for the functional age gap severity classification task. The selected model [35] used entropy as the splitting criterion, a maximum depth of 3, a minimum of 10 samples required to split an internal node, a minimum of 2 samples required at each leaf node, balanced class weights, and 300 trees in the final ensemble. Model performance was evaluated using repeated stratified k-fold cross-validation with 6 folds and 10 repetitions.

### 2.4. Feature Importance

We also reported the contribution of the features in the prediction through the SHapley Additive exPlanations (SHAP) technique. SHAP methods assign a score to each input feature based on its influence on the target variable. In the context of ML, SHAP values assign each feature an importance score for a specific prediction, offering insights into how each feature impacts the model’s output. To improve interpretability, SHAP values were computed for the ensemble model. Explanations were generated for each tree estimator and then averaged across the ensemble to obtain a global ranking of feature importance.

## 3. Results

### 3.1. Descriptive Statistics

**Figure 1:**
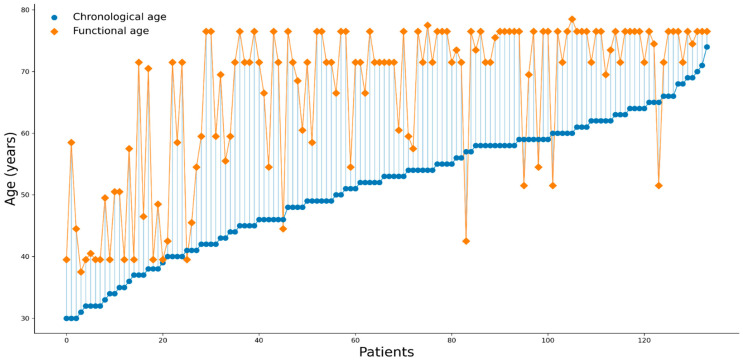
Comparison between chronological age and functional age in the AKU cohort. Circles represent chronological age, and diamonds represent functional age. Vertical lines indicate the magnitude and direction of the functional age gap.

A total of 134 patients with AKU were included in the analysis. The mean chronological age of the cohort was 51 years (median 53; range 30–74), whereas the mean functional age was 66 years (median 71.5; range 37–78). Accordingly, the functional age gap had a mean value of 15 years, with a median of approximately 15 years and an interquartile range of 8–20 years. Its broad distribution indicated marked inter-individual variability in functional impairment. Although a small subgroup of patients showed negative values, corresponding to a functionally younger profile than expected for their chronological age, the vast majority exhibited positive gaps. Indeed, in 127 of 134 patients (94.8%), functional age exceeded chronological age, indicating that premature functional decline was the predominant pattern in the cohort. To further examine this pattern, chronological and functional age were compared for each patient, as shown in Figure 1.

In most cases, functional age was higher than chronological age, and the distance between the two measures reflected the magnitude of the discrepancy for each patient. This divergence was observed across the entire age spectrum, suggesting that individuals of similar chronological age could exhibit markedly different functional profiles. Notably, many individuals in mid-adulthood showed a functional profile more consistent with that of considerably older age groups.

**Figure 2:**
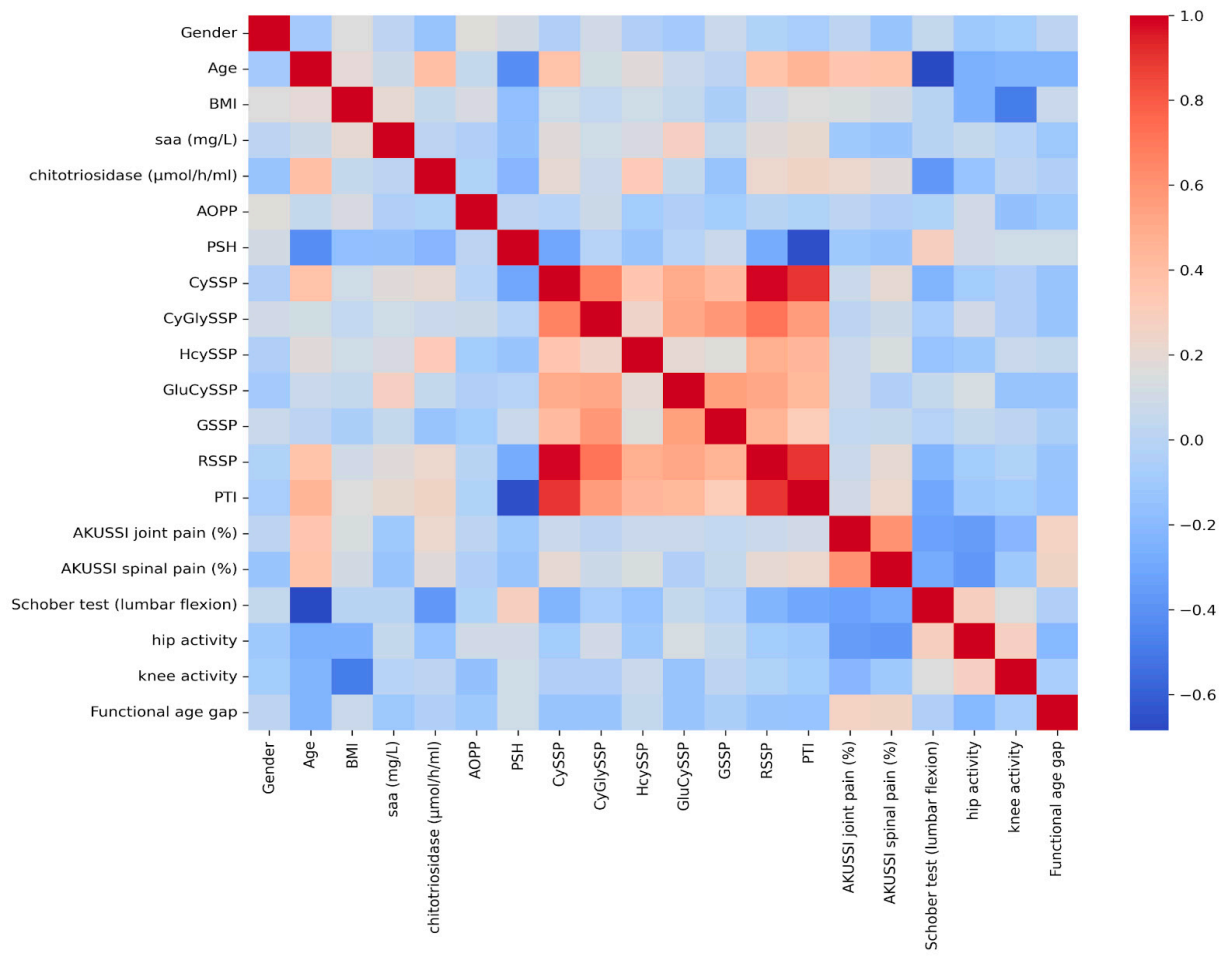
Correlation matrix between the functional age gap and demographic, biochemical, and clinical variables. Color intensity represents the strength and direction of the correlation coefficient (r), with positive correlations shown in red and negative correlations in blue.

A correlation analysis was performed based on the Pearson correlation coefficient to assess the linear association between the functional age gap and the available demographic, biochemical, and clinical variables. The results are summarized in Figure 2. The analysis showed only weak-to-moderate associations, with no evidence of strong linear relationships between the functional age gap and any individual variable. The highest positive correlations were observed for AKUSSI joint pain (r = 0.278) and AKUSSI spinal pain (r = 0.275), which suggested that greater pain burden was associated with a larger discrepancy between functional and chronological age. Among the negative correlations, the strongest were found for hip activity (r = −0.239) and chronological age (r = −0.215), indicating that reduced hip function and younger age were associated with a higher functional age gap.

These findings indicate that no single variable exhibits a strong linear association with the functional age gap. This suggests that individual bivariate relationships provide only limited explanatory power and supports the use of multivariable machine learning models, which may capture more complex dependencies among the available predictors.

### 3.2. Model Performance and SHAP Analysis

To investigate whether functional age gap severity could be predicted from the available multivariable information, a bagging ensemble classifier based on decision trees was applied to the three severity classes derived from the functional age gap. This model was selected after comparison with baseline classifiers (XGBoost, decision tree, Support Vector Machine (SVM), and K-Nearest Neighbors (KNN)) evaluated under the same repeated stratified cross-validation scheme. The bagging ensemble achieved the highest mean accuracy (0.64 ± 0.08) and macro F1-score (0.61 ± 0.09), outperforming XGBoost (accuracy 0.59 ± 0.09; macro F1 0.58 ± 0.09), decision tree (0.63 ± 0.10; 0.59 ± 0.10), SVM (0.53 ± 0.10; 0.52 ± 0.10), and KNN (0.45 ± 0.09; 0.44 ± 0.10). These values are consistent with what can reasonably be expected from an exploratory multiclass classification task in a rare-disease cohort of limited size, and should not be interpreted as evidence of a clinically deployable individual-level predictor. Rather, they indicate that the three severity classes of the functional age gap are organised according to coherent multivariable patterns that the model can partially recover.

To further characterize the classification errors, we computed the confusion matrix averaged across repeated stratified cross-validation folds, as shown in Table 1.

The matrix showed that the low and high functional age gap classes were identified more reliably, with diagonal values of 0.67 and 0.83, respectively. In contrast, the intermediate class showed lower discrimination, with a diagonal value of 0.37, and was distributed across both neighbouring classes. Overall, this pattern suggests that the model does not fail broadly across all severity categories, but rather that the intermediate severity class partially overlaps with the low and high classes, as expected in an ordinal severity classification setting.

**Figure 3:**
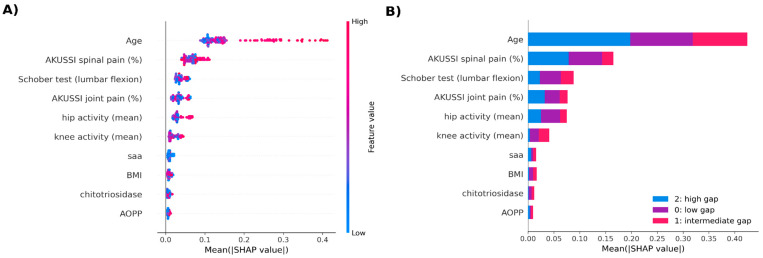
SHAP-based interpretation of the functional gap classification model. (**A**) Global SHAP summary plot of the top 10 predictors, ranked by mean absolute SHAP value. Dot colour represents the feature value, from low to high. (**B**) Class-specific SHAP bar plot showing the contribution of each feature to the prediction of low, intermediate, and high functional gap classes.

The interpretability of the bagging ensemble classifier was improved by applying SHAP analysis, which quantified the contributions of individual variables to predicting functional age gap severity. Global feature importance was defined as the mean absolute SHAP value, computed by averaging SHAP contributions over all samples and all estimators in the ensemble. To further explore whether predictor relevance differed across severity levels, class-specific SHAP values were also computed for the low, intermediate, and high functional age gap classes. The results are summarized in Figure 3.

As expected, age ranked as the most influential variable in the global SHAP analysis, showing the highest mean absolute SHAP value and the broadest distribution of patient-level contributions. AKUSSI spinal pain (%) and AKUSSI joint pain (%) were also identified as major contributors, confirming the central role of pain-related measures in explaining functional impairment. In addition, the Schober test (lumbar flexion) showed a relevant contribution, highlighting the importance of spinal mobility in the prediction of functional decline. Measures of joint function, including hip activity and knee activity, also ranked among the most relevant features, further supporting the role of reduced mobility in determining functional age gap severity. Biochemical markers, including SAA, chitotriosidase, and AOPP, contributed to model predictions to a lesser extent, while BMI showed a smaller but measurable contribution within the overall feature importance profile. Class-specific SHAP analysis further refined this interpretation. The multiclass bar plot showed that age remained the dominant predictor across low, intermediate, and high functional age gap classes, with the strongest contribution observed for the high-gap category. In contrast, AKUSSI spinal pain, Schober test, AKUSSI joint pain, hip activity, and knee activity displayed class-dependent patterns, indicating that pain and mobility-related variables contribute differently to the discrimination among severity groups.

To confirm that the model’s predicted structure was not solely influenced by chronological age, a sensitivity analysis was conducted. This involved refitting the bagging ensemble after excluding chronological age from the predictor set, while maintaining all other features, hyperparameters, and the cross-validation scheme. The model excluding age showed reduced classification performance, with mean accuracy, macro F1-score, and macro recall of 0.42 ± 0.09, 0.41 ± 0.09, and 0.42 ± 0.09, respectively. This confirms that chronological age substantially contributes to the multivariable separation of the severity classes, which is consistent with its dominant ranking in the SHAP analysis. It also reinforces the decision to base the biological interpretation of the model primarily on non-age predictors: AKUSSI spinal and joint pain, the Schober test and hip and knee activity.

## 4. Discussion

This study introduces the concept of the functional age gap in AKU as a quantitative and clinically interpretable measure of the discrepancy between chronological age and functional status. A relevant contribution of this study lies in the integration of different patient-reported functional measures into a single interpretable framework. HAQ-DI and KOOS capture partially distinct dimensions of impairment. HAQ-DI reflects general disability in everyday activities, whereas KOOS focuses more specifically on knee-related symptoms, pain, function, and quality of life. Treating these instruments as isolated endpoints may therefore fragment the representation of patient burden. In contrast, the functional age approach translates both into a common age-related metric and combines them into a unified estimate of biological and functional burden. This does not eliminate the conceptual differences between the two questionnaires, but it allows them to be interpreted jointly within an age-referenced framework that is intuitively meaningful and clinically accessible, important in the context of AKU-specific precision medicine. Our findings identify that most patients in the cohort displayed a functionally older profile than expected for their chronological age. This supports the view that premature functional decline is a core feature of AKU and provides a novel framework for capturing disease burden at the individual level [14]. From a pathophysiological perspective, this observation is coherent with the known natural history of AKU [10,12,13]. The lifelong accumulation of HGA and the subsequent development of ochronosis progressively damage connective tissues, cartilage, and weight-bearing joints, ultimately leading to early musculoskeletal deterioration. The predominance of positive functional age gaps in our cohort suggests that this decline may become evident before, or independently of, the full expression of the typical clinical manifestations described from early adulthood onwards [36]. In this sense, the functional age gap not only confirms that AKU is disabling but also quantifies the extent to which disability differs from what would be expected based on age alone. Another important result of this study is the marked inter-individual variability observed across patients of similar chronological age. Although functional deterioration was widespread, its magnitude differed substantially between subjects, indicating that AKU follows heterogeneous functional trajectories. This is consistent with previous evidence showing that disease severity and progression cannot be adequately explained by genotype or by isolated clinical features alone [22,23]. Within this framework, the functional age gap can be interpreted as an emergent systems-level phenotype that reflects the integrated effect of these multiple disease dimensions.

This interpretation is further supported by correlation analysis, which shows weak-to-moderate bivariate linear associations between the functional age gap and the available variables. The limited bivariate signal, together with the moderate but stable performance of the multivariable bagging classifier, is consistent with a scenario in which functional impairment in AKU emerges from the combined contribution of several factors, possibly involving non-linear dependencies, although the available cohort size does not allow this hypothesis to be tested formally. In this context, the performance of the ML model indicates that functional impairment in AKU is structured according to multivariate patterns that only emerge when the variables are considered together. Such an approach is especially relevant in rare diseases, where relatively small cohorts and heterogeneous phenotypes often limit the explanatory power of conventional statistical models [37,38]. The SHAP analysis further strengthens the biological and clinical plausibility of the model. Pain-related variables, particularly AKUSSI spinal pain and AKUSSI joint pain, were among the most influential features, confirming the central role of chronic pain in driving disability and reduced quality of life in AKU. Measures of mobility and musculoskeletal function, including hip activity, knee activity, and the Schober test, also contributed substantially to prediction, highlighting how cumulative damage to large joints and spinal flexibility translates into impaired daily functioning and greater patient-reported disability.

By contrast, inflammatory and oxidative-stress biomarkers contributed more modestly to model predictions. This should not be interpreted as evidence that these pathways are unimportant in AKU. On the contrary, previous studies have reported altered inflammatory and redox-related markers, including SAA, chitotriosidase activity, and oxidative stress parameters, in subsets of patients with AKU, with possible links to biological stress and disease severity. However, these variables may be more informative about underlying molecular activity or long-term tissue damage than about cross-sectional functional status at the individual level. In other words, they may participate in disease progression without necessarily acting as dominant standalone surrogates of immediate disability. From a clinical perspective, the functional age gap may represent a candidate intermediate digital endpoint that warrants further investigation. Because it condenses multidimensional functional information into a single age-referenced measure, it may support patient stratification and help identify subjects with unexpectedly severe impairment. However, the moderate classifier performance and the limited cohort size preclude conclusions on individual-level predictive utility. Therefore, these findings should be considered hypothesis-generating and require validation in larger and longitudinal cohorts. Pending such validation, the functional age gap may also prove useful for assessing therapeutic response or enriching patient subgroups in future interventional studies. In particular, identifying patients whose functional profile appears substantially older than their chronological age could reveal clinically relevant deterioration that is not yet fully reflected by traditional severity indices alone.

Several limitations should nevertheless be acknowledged. First, the sample size remains limited, which is an intrinsic challenge in AKU research and may restrict the generalizability of the model. Second, the derivation of functional age from normative reference tables introduces dependence on external populations and may not fully account for sex-, culture-, or cohort-specific differences in questionnaire behavior. Specifically, HAQ-DI reference values were derived from a Central Finnish general-population sample [30], whereas KOOS reference values were derived from a Danish national sample [29], while the present AKU cohort is Italian. Moreover, these references describe general-population functioning rather than AKU-specific or disease-specific trajectories. Therefore, the functional age gap should be interpreted as the deviation of AKU patients from age-expected general-population functioning, rather than as an absolute estimate based on an AKU-specific benchmark. Because no Italian or AKU-specific normative data are currently available, these references provide a pragmatic standard, but the absolute magnitude of the estimated gap should be interpreted with caution. Third, while this cross-sectional analysis establishes the functional age gap as a tool for patient stratification and clinical interpretation, its role as a longitudinal biomarker remains to be fully characterized. An additional methodological limitation concerns the possible circularity introduced by chronological age. Therefore, model interpretation should focus primarily on non-age-related predictors, such as pain-related measures, spinal mobility, and joint function, whose relevance is biologically consistent with AKU pathophysiology. Future work should focus not only on expanding the dataset but also on improving the biological and clinical context in which the functional age gap is interpreted. Longitudinal integration of patient-reported outcomes with treatment exposure, imaging, biomechanical parameters, and molecular markers could provide a more complete representation of AKU progression and help clarify the mechanisms underlying functional decline. More broadly, AKU may represent an informative model for investigating how chronic endogenous metabolic stress contributes to degeneration, impaired resilience, and aging-like functional decline. Within this broader framework, the functional age gap could evolve from a descriptive metric into a clinically informative tool for patient stratification and personalized disease monitoring.

## 5. Conclusions

In this study, we introduced the concept of functional age gap as a novel metric to assess functional impairment in AKU. Our findings demonstrate that premature functional decline is a predominant feature of AKU, with most patients exhibiting a functionally older profile than expected. The functional age gap, therefore, represents a promising integrative descriptor, with potential applications in patient stratification and, pending longitudinal validation, in disease monitoring and personalized management. Future work should focus on validating this metric in larger and longitudinal cohorts, as well as exploring its utility in evaluating treatment response and guiding precision medicine approaches in AKU.

## Figures and Tables

**Table 1:** Confusion matrix averaged across repeated stratified cross-validation folds. Rows indicate the true functional age gap severity classes, whereas columns indicate the predicted classes. Values represent the average fraction of patients from each true class assigned to each predicted class. Diagonal values indicate correct classifications.

	Predicted Class 0	Predicted Class 1	Predicted Class 2
**Class 0**	0.67	0.15	0.17
**Class 1**	0.29	0.37	0.35
**Class 2**	0.11	0.06	0.83

## Data Availability

The raw data supporting the conclusions of this article will be made available by the authors on request.

## Supplementary Materials

The following supporting information can be downloaded at: https://www.mdpi.com/article/10.3390/bioengineering13060604/s1, Table S1: List and description of the clinical, biochemical, oxidative stress, functional, and disease-severity variables included in the study.

## Abbreviations

The following abbreviations are used in this manuscript:

AKU	Alkaptonuria
HGD	Homogentisate 1,2-dioxygenase
HGA	Homogentisic acid
QoL	Quality of life
HAQ-DI	Health Assessment Questionnaire Disability Index
KOOS	Knee Injury and Osteoarthritis Outcome Score
AKUSSI	Alkaptonuria Severity Score Index
BMI	Body mass index
SAA	Serum amyloid A
AOPP	Advanced oxidation protein products
PTI	Protein thiolation index
PSH	Protein thiols
CySSP	Cysteinylated proteins
CyGlySSP	Cysteinylglycinylated proteins
HcySSP	Homocysteinylated proteins
GluCySSP	Glutathionylated cysteinylated proteins
GSSP	Glutathionylated proteins
RSSP	Total protein thiolation-related species
ML	Machine learning
AI	Artificial intelligence
SHAP	SHapley Additive exPlanations
SVM	Support Vector Machine
KNN	K-Nearest Neighbors
